# Genetic Sentilations

**Published:** 1915-11

**Authors:** 


### Genetic Sentilations.

It is the purpose of this department to stand for the truth as we see it, and in no sense to be a partisan on any question. We are, therefore, open to conviction and we welcome any suggestion that seems worthy of scientific consideration. So long as a question is based on a theoretical conception it is open to revision, and it is only by constantly attacking and testing its premises that the truth can eventually be known. The past few years have been most fruitful in the production of new facts on questions of genetics and there has arisen quite a friendly rivalry between the psychologist's and biologist's viewpoints. The balance of proof has been largely in favor of the biologist with his theories of heredity and mutitions. Now comes the psychologist's proofs that may compel some modifications of his opponent's dictum. From his study of unconscious phenomena, the psychologist is convinced that many traits of the adult are due to impressions on early life rather than to heredity. He believes that in the matter of character and personality, to assume that certain peculiarities are due to the presence or absence of specific determiners are not substantiated by actual facts. In controversion of this theory, one of these psychologists, Dr. Samuel C. Kohs, before the American Genetic Association at Berkeley, in August of this year, again brought into question the inheritance of acquired characters. He quotes Semon's mnemic theory, which claims "that ontogeny is a mnemic phenomenon, evolution depending on a change in ontogenetic rhythm." According to this view nerve cell and germ cell both possess the property of being impressionable, the latter, of course, to a much lesser degree. The engram is the unit of impression, many of these being associated into various different groups, the whole completed system being called the "mneme," a Greek term equivalent to "memory." It is claimed that experiences, habits, will leave their traces upon the organism, the germ plasm only being fundamentally affected after a vast number of reinforced repetitions. The "imprint" having been made, later generations will show changes either in structure or function."

He also quotes the observing remarks of Jennings, who said: "As a material, potentially visible organism, I, like the infusorian, have been in existence ever since the race that developed into human kind began. And this, for each of us, is not a figure of speech, but the plain literal truth. An unlimited microscopist could have followed with his eyes my course, and your course, down through countless ages, never losing sight of the material organism for an instant. * * * I was in actual material existence as a living organism, and indeed thousands or millions of years old, when the pyramids were built, and my unlimited microscopist could give my history from that time to this without a break. What marks has that long history left on my personality and character?"

This same author points out what he regards as a weak spot in the positive eugenic program by the inclusion of certain mental, emotional and moral traits in the category of inheritable units. He argues this on the grounds of unconscious activity in that by for the major portion of our mental life nerve enters the realm of consciousness. "We would all be sad wrecks in but a very short space of time," he reasons, "were all the mental activity of each successive moment to project itself into our conscious field."

The author illustrates his point by referring to Freud and his doctrine. "The Freudians," he says, "and those closely allied to them have uncovered a large number of mental mechanisms whose roots lie in the subconscious. Data first obtained in gross form from those mentally disturbed were found to apply quite as well to people whose psyche was in normal activity. And the great contribution of the Zurich school to the psychology of today has been the discovery of the existence of the complex and the explanation of how it functions. Previous to this time all we could do was just to make disconnected observations such as that of Le Bon in his 'Psychology of the Crowd': 'Behind the avowed causes of our acts there undoubtedly lie secret causes that we do not avow, but behind these secret causes there are many others more secret still, which we ourselves ignore. The greater part of our actions are the result of hidden motives which escape our observations.' But now we know that the complex, once it is formed, becomes a potent, dynamic unit, and that some of these 'blind impulses to action' are merely the inevitable result of its existence.

"A complex, simply defined, is an associative arrangement of specific mental data, strongly tinged emotionally. Complexes may exist in the realm of the conscious, but are of as great, if not of greater significance, when they sink below the limit of consciousness. Education, environment, home influences, exceptional experiences, age, sex, race, religion, certain hereditary predispositions, are among the most important factors determining the type, number and trend of one's complexes. The recent research of psychoanalysts, whose testimony and data ought not to be ignored, has indicated the close and intimate relationship between such phenomena as forgetting, moods, character, likes and dislikes, ambitions, mental and physical ability, habits, the development of special aptitudes,—and the complex. The analyses of insane persons and criminals, on the one hand, and those of geniuses, artists, poets, on the other, have shown that the type of complex in both groups is very much alike. These dynamic units, by the way, find their counterpart in the engrams of Semon's mneme, but it does not necessarily follow from that that all complexes are inherited; some are, a large number are not."

Thus the psychologist interprets certain acts as being the expression of the subconscious ego rather than to heredity. The Zurich school holds that the principal roots of personality and character lie in the unconscious, and that the early impressions of childhood were of enormous significance in the later development of the individual. Hence what has been mistaken for heredity is the impress of the parent on the unconscious child.

Adler says: "None of us is born with a system all of whose organs are perfect in structure or function. There is a weak spot somewhere. This will be manifested very early in life in defective reactions to the environment. An attempt, conscious or unconscious, will then be made to compensate for that defect, either by a symmetrical organ undertaking the extra labor, or by an entirely different organ adapting itself to care for the extra burden, the defective organ in both cases becoming hypertrophied; or, finally, by a hyperfunctioning of the inferior organ. This compensation activity may also be manifest in cases where organ-defect is not necessarily the basis. For example, a child lies about the occupation of its father, saying that he drives the king's carriage, when in reality he is a coal dispenser in very poor circumstances. The defect here is, of course, one of social position, and the extra activity of the imagination is induced to compensate for this defect."

Thus the psychologist believes that a trait not specially desired by the individual need not be carried on through his life, but, by a proper handling of environmental, unconscious, and hereditary factors, such harmful traits can be corrected, "sublimated," and the energy diverted into better direction.

The biologist is very convincing in his demonstrations, yet, the psychologist has the advantage in the facts of experience. Let us have both as the truth is hurt by neither and may be that out of the "burnishing" will evolve a better understanding.

